# Probiotics Inhibit Cartilage Damage and Progression of Osteoarthritis in Mice

**DOI:** 10.1007/s00223-022-01030-7

**Published:** 2022-10-19

**Authors:** Antonia Sophocleous, Asim Azfer, Carmen Huesa, Eleni Stylianou, Stuart H. Ralston

**Affiliations:** 1 Department of Life Sciences, School of Sciences, European University of Cyprus, Nicosia, Cyprus; 2 Rheumatology and Bone Diseases Unit, Centre for Genomic and Experimental Medicine, Institute of Genetics and Cancer, Western General Hospital, University of Edinburgh, Edinburgh, UK; 3 Institute of Infection, Immunity & Inflammation, College of Medical, Veterinary and Life Sciences, University of Glasgow, Glasgow, UK

**Keywords:** Microbiome, Probiotics, DMM, Osteoarthritis

## Abstract

Increasing interest has focussed on the possible role of alterations in the microbiome in the pathogenesis of metabolic disease, inflammatory disease, and osteoporosis. Here we examined the role of the microbiome in a preclinical model of osteoarthritis in mice subjected to destabilisation of medical meniscus (DMM). The intestinal microbiome was depleted by broad-spectrum antibiotics from 1 week before birth until the age of 6 weeks when mice were subjected reconstitution of the microbiome with faecal microbial transplant (FMT) followed by the administration of a mixture of probiotic strains *Lacticaseibacillus paracasei* 8700:2, *Lactiplantibacillus plantarum* HEAL9 and *L. plantarum* HEAL19 or vehicle. All mice were subjected to DMM at the age of 8 weeks. The severity of osteoarthritis was evaluated by histological analysis and effects on subchondral bone were investigated by microCT analyses. The combination of FMT and probiotics significantly inhibited cartilage damage at the medial femoral condyle such that the OARSI score was 4.64 ± 0.32 (mean ± sem) in the FMT and probiotic group compared with 6.48 ± 0.53 in the FMT and vehicle group (*p* = 0.007). MicroCT analysis of epiphyseal bone from the femoral condyle showed that the probiotic group had higher BV/TV, increased Tb.Th, and moderately thicker subchondral bone plates than the control group. There was no difference between groups in joint inflammation or in serum concentrations of inflammatory cytokines and chemokines. We conclude that treatment with probiotics following FMT in mice where the microbiome has been depleted inhibits DMM-induced cartilage damage and impacts on the structure of subchondral bone particularly at the femoral condyle. While further studies are required to elucidate the mechanism of action, our research suggests that these probiotics may represent a novel intervention for the treatment of osteoarthritis.

**Supplementary Information:**

The online version contains supplementary material available at 10.1007/s00223-022-01030-7.

## Introduction

Osteoarthritis (OA) is the most common cause of disability in older people. The underlying cause is incompletely understood, and medical management is based on trying to control pain.

Over the past few years increasing interest has focussed on the role of the microbiome in the pathogenesis of various diseases including inflammatory disease, osteoporosis, and OA [1–3]. In 2018, Schott and colleagues showed that obesity-related dysbiosis of the gut microbiome leads to osteoarthritis of obesity [4]. A population-based cohort study, looking at the gut microbial composition of 1427 participants showed that there was an association between composition of the gut microbiome, knee pain and evidence of knee inflammation as assessed by MRI [3]. Over recent years, numerous randomised clinical trials have been performed to investigate the effects of probiotics in a variety of diseases, affecting the gastrointestinal system [5]. Additionally, one randomised placebo-controlled trial reported that the probiotic *Lactobacillus casei Shirota* improved pain in patients with knee osteoarthritis [6]. There is pre-clinical evidence that probiotics have anti-inflammatory effects and protect against ovariectomy-induced bone loss [7] and that they restore microbiome dysbiosis to protect bones from destruction in a rat model of rheumatoid arthritis [8].

To further evaluate the role of the microbiome the pathogenesis of OA we investigated the effects of antibiotic induced ablation of the microbiome followed by reconstitution and the administration of probiotics in a mouse model where OA is surgically induced by destabilisation of the medial meniscus (DMM). The main aim of this study was to investigate whether ablation of the microbiome followed by microbiome reconstitution together with probiotics administration influence the development of OA.

## Materials and Methods

### Materials

Antibiotics were obtained from Sigma Aldrich (Dorset UK). PBS was obtained from Invitrogen (Paisley, UK). Probiotics and glycerol were kindly provided by Probi AB (Lund, Sweden). The Immunology Multiplex Assay MILLIPLEX® Mouse Cytokine/Chemokine Magnetic Bead Panel was purchased from Merck (UK).

### Experimental Design

**Fig. 1:**
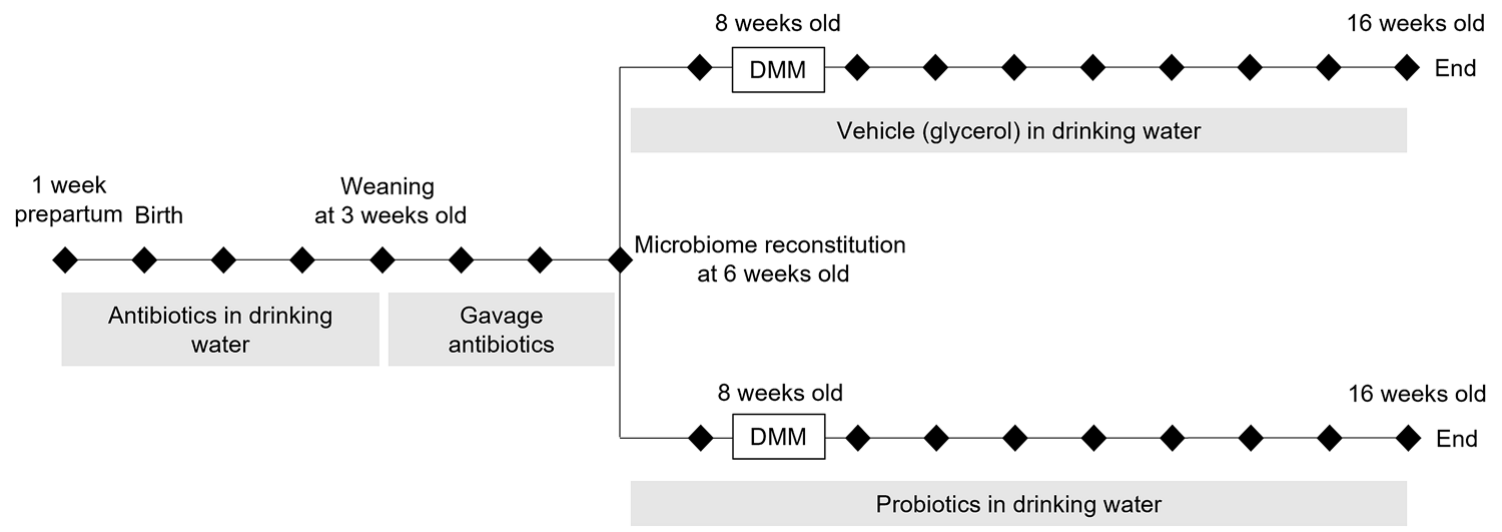
Overview of experimental design

The experiment was carried out using 21 male C57BL/6 mice, which were housed in groups of 3–5 per cage in pathogen-free rooms of a designated animal facility, at constant temperature, under a 12-h dark-to-light cycle with water and pelleted standard commercial diet made available ad libitum. An overview of the experimental design is shown in Fig. 1. The microbiome was disrupted in all mice by administration of antibiotics to the parent from 1 week before birth until weaning and subsequently by gavage in the offspring until the age of 6 weeks as previously described [9, 10]. This was achieved by administration of ampicillin in drinking water (1 g/L) from 1 week before birth until mice reached the age of 3 weeks. Subsequently, an antibiotic cocktail consisting of Vancomycin 5 mg/ml, Neomycin 10 mg/ml, Metronidazole 10 mg/ml and Amphotericin B 0.1 mg/ml was administered daily by gavage for 3 weeks as previously described [10].

All mice were subjected to reconstitution of the intestinal microbiome by faecal microbiota transplantation (FMT). This was achieved by administering faecal samples from healthy mice to the recipient mice [9]. The donor faecal samples were collected using aseptic technique and diluted 1:10 in a 50% glycerol/PBS solution, frozen in liquid nitrogen and kept in – 80 °C until use. On the day of FMT, the faecal solution was thawed and diluted 1:5 in Glycerol/PBS and 150 mL was administered via oral gavage to each recipient mouse.

### Probiotic Treatment

Following FMT, mice were treated with a mixture of probiotic strains *Lacticaseibacillus paracasei* 8700:2 (DSM13434), *Lactiplantibacillus plantarum* HEAL9 (DSM 15,312) and *Lactiplantibacillus plantarum* HEAL19 (DSM 12,313) in equal amounts (*n* = 11), or vehicle (glycerol) (*n* = 10) for a period of 10 weeks. The probiotic or vehicle treatment began 2 weeks before destabilisation of medial meniscus (DMM) and continued for 8 weeks following surgery. The strains *Lacticaseibacillus paracasei* 8700:2 (DSM13434), *Lactiplantibacillus plantarum* HEAL9 (DSM 15,312) and *Lactiplantibacillus plantarum* HEAL19 (DSM 12,313) were administered in drinking water at a concentration of 10^9^ colony-forming units (CFU)/ml. Water bottles with probiotic mixture were changed daily. Although survival of the probiotic bacteria was not assessed in this experiment, evidence from other studies showed that their concentration in drinking water drops one log unit per day, to approximately 10^8^ CFU/ml [7, 11].

### Destabilisation of the Medial Meniscus

The mice were subjected to destabilisation of medial meniscus (DMM) as described by Glasson et al. [12]. Following general anaesthesia, the medial meniscotibial ligament of the knee was severed using a scalpel or 2 mm blade spring scissors, and the joint capsule and skin were subsequently closed. Sham surgery was not performed on the contralateral knee based on animal welfare grounds since previous studies had shown no difference in OA scores between sham operated and non-operated knee joints using this model [12]. Mice were followed up for 8 weeks post DMM and sacrificed at age 16 weeks by inhalation of carbon dioxide. The lower limbs were dissected, fixed for 24 h in 4% formaldehyde and then kept in ethanol (70% v/v) until further analysis. Knee joints were then phenotyped for evidence of OA by MicroCT analysis to look for quantitative and qualitative changes in subchondral bone and by histological examination of cartilage with quantitation of severity according to the Osteoarthritis Research Society International (OARSI) guidelines [13].

### Micro Computed Tomography

Analysis of periarticular bone was performed by micro-computed tomography (microCT) using a Skyscan 1172 instrument set at 60 kV and 167 mA, at a resolution of 5 μm. The regions of interest analysed were the subchondral trabecular bone situated within the tibial and femoral epiphysis and the subchondral bone plates of the tibial plateau and femoral condyle. MicroCT analyses were performed in the coronal plane. Following acquisition, the images were reconstructed using the Skyscan NRecon programme and analysed using the Skyscan CTAn software.

### Histology and Assessment of OA Severity

Histological analysis was performed on fixed whole joints that had been decalcified in 10% formic acid for 1 week. The joints were then processed and embedded in paraffin wax according to standard techniques. A Leica microtome (Solms, Germany) was used to take 7 μm coronal sections through the entire joint at 45 μm intervals, yielding 10–13 different levels. Sections were then stained with Safranin-O and Haematoxylin according to standard techniques. Histological evaluation of the severity of osteoarthritis and inflammation was performed by two observers blinded to intervention according to the OARSI scoring system [13] and the semi-quantitative synovitis scoring system [14], respectively.

Reproducibility of the OARSI scoring system yielded an interobserver kappa coefficient of 0.66, which is considered substantial. Reproducibility of the synovitis/inflammation scoring system yielded a kappa coefficient of 0.86, which is considered almost perfect agreement [15].

The OA scores were generated separately from the medial tibial plateau (MTP), the medial femoral condyle (MFC), the lateral tibial plateau (LTP) and lateral femoral condyle (LFC) for each section evaluated. The severity of OA was expressed as summed scores at each site of the joint for all the sections evaluated. Scores for three parameters at the medial compartments of the knee joints, pannus formation, thickening of the synovial membrane (synovial hyperplasia), and sub-synovial hyperplasia, were generated for all the sections evaluated. The synovitis/inflammation severity was expressed as the average of summed scores of the three parameters across all sections evaluated.

### Multiplex Cytokine Bead Array Assay

Samples of mouse serum were diluted 1:3 in assay buffer and analysed by MILLIPLEX® MAP Mouse Cytokine/Chemokine Magnetic Bead Panel (MCYTMAG70PMX25BK, Merck Millipore), following the manufacturer’s instructions. Briefly, the wells of the 96 well plate kit were filled in duplicate with 25 µl each of standards, control, and the samples. This followed by the addition of 25 µl beads to each well. The beads used were pre-coated with monoclonal antibodies specific for a single cytokine or chemokine and were internally labelled with fluorescent dyes. The plate was incubated overnight (2–8 °C) followed by removal of contents from the wells and rinsed twice with buffer to remove the unbound proteins. Next, 25 µl of biotinylated detection antibodies were added in each well and incubated for an hour at room temperature followed by 30 min at room temperature following the addition of 25 µl streptavidin–phycoerythrin (Strep-PE). Finally, after washing the unbound proteins, the wells were filled with sheath fluids (150 µl). The plate was run under Luminex® 200™ system to measure the concentration of cytokines/chemokines in mouse serum. The data were saved and analysed for Median Fluorescent Intensity (MFI) using a 5-parameter logistic curve-fitting method.

### Statistical Analysis and Sample Size

Statistical analyses were performed using IBM SPSS Statistics, version 25 (Armonk, NY). Required sample size to provide 85% power to detect a 1.2 standard deviation difference in severity of osteoarthritis between two treatment groups was calculated using G*Power 3.1.9.7 [16]. The inter-observer agreement between the OARSI scores of AS and ES was assessed by calculating the kappa coefficient. For all experiments we determined if the data were normally distributed by calculating skewness and kurtosis. For data that were normally distributed, between-group comparisons were performed by independent-samples *T* test analysis. For data that were not normally distributed, comparisons were performed using the Mann–Whitney *U* test. Unless otherwise stated the values shown are the mean and standard error of mean (SEM). The significance level was set at *p* ≤ 0.05 for individual experiments, except for the multiplex cytokine bead serum array where the significance level was adjusted to *p* = 0.002 to take account of multiple testing.

## Results

### Effect of Microbiome Depletion and Probiotics on Cartilage Damage

**Fig. 2:**
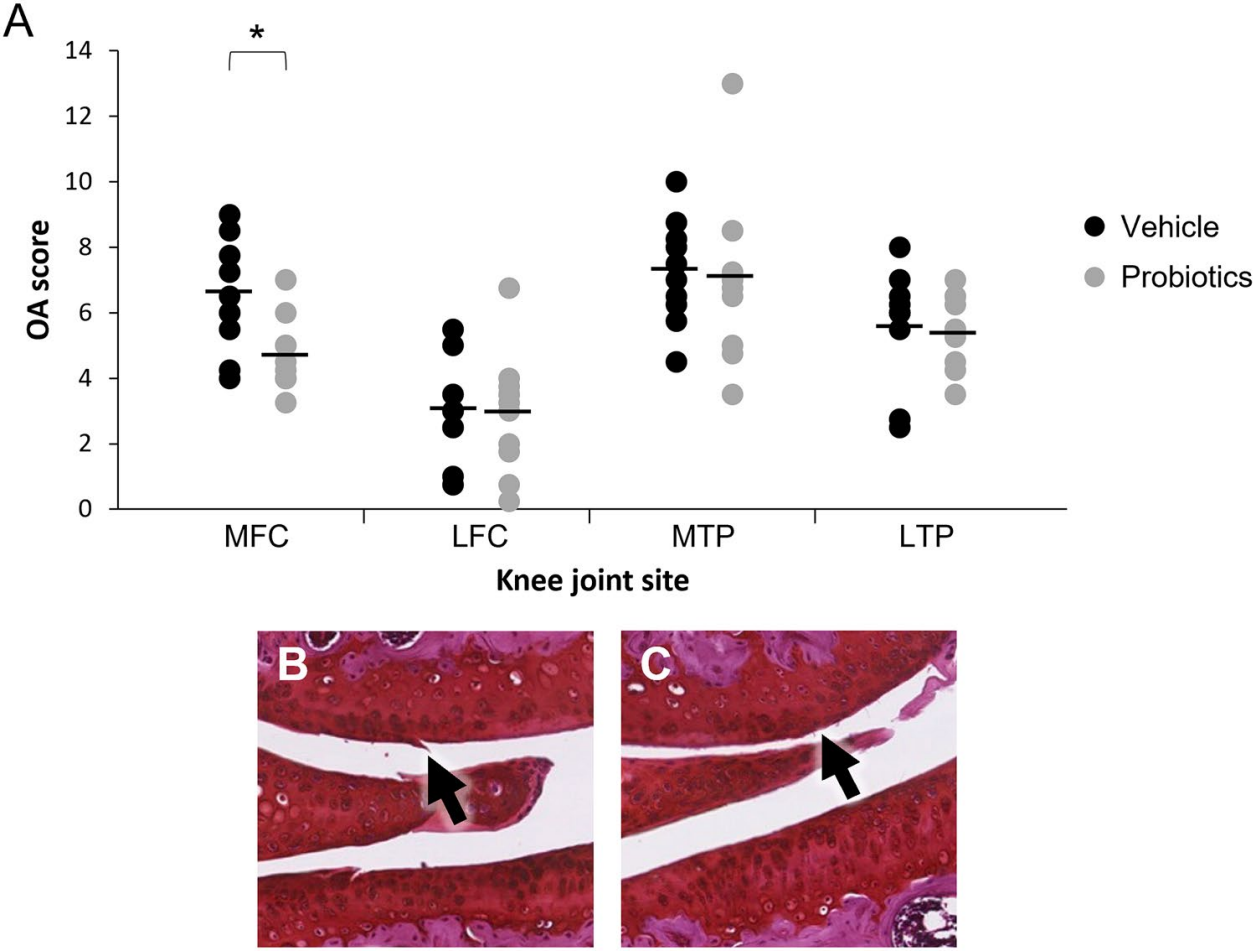
Probiotics reduce cartilage damage at the medial femoral condyle. **A** Cartilage damage scores assessed by the OARSI system at the Medial Femoral Condyle (MFC); the Medial Tibial Plateau (MTP); the Lateral Femoral Condyle (LFC); and the Lateral Tibial Plateau (LTP) of DMM-operated knee joints from two different treatment groups, vehicle (*n* = 10) and probiotics (*n* = 11). The results here are the sum of all OA scores across the knee joints. Each horizontal line indicates the mean value for each respective joint site and the circles represent individual values (*p < 0.007 between groups). **B** and **C** Representative photomicrograph from the medial femoral condyle (MFC) of DMM-operated knee joints in mice from the two different treatment groups. **B** shows the MFC with a score of two from a mouse in the vehicle treatment group with a vertical cleft in the superficial layer of cartilage (arrow). **C** shows the MFC with a score of one from a mouse in the probiotic treatment group. There are small cartilage surface fibrillations without cartilage loss

There was no significant difference in OARSI cartilage damage scores for medial tibial plateau (MTP) or lateral tibial plateau (LTP) between the groups (Fig. 2). The OARSI scores at the medial femoral condyle (MFC) were significantly lower in mice who had undergone FMT and received probiotics treatment compared to the control group (FMT only). Values for OARSI scores with probiotics treatment were (mean ± sem) 4.64 ± 0.32 compared to 6.48 ± 0.53 in mice with vehicle treatment, representing a percentage difference of 28.4 ± 5.0% (*p* = 0.007) (Fig. 2).

Representative images of cartilage damage at the MFC of DMM-operated joints in the two treatment groups are shown in Fig. 2B and C. The image from a mouse randomised to vehicle treatment shows a score of 2 with clefts immediately below the superficial layer (Panel B) and the image from a mouse randomised to probiotics treatment shows a score of 1, with only superficial cartilage surface fibrillations evident (Panel C).

### Effect of Microbiome Depletion and Probiotics on Subchondral Bone

**Fig. 3:**
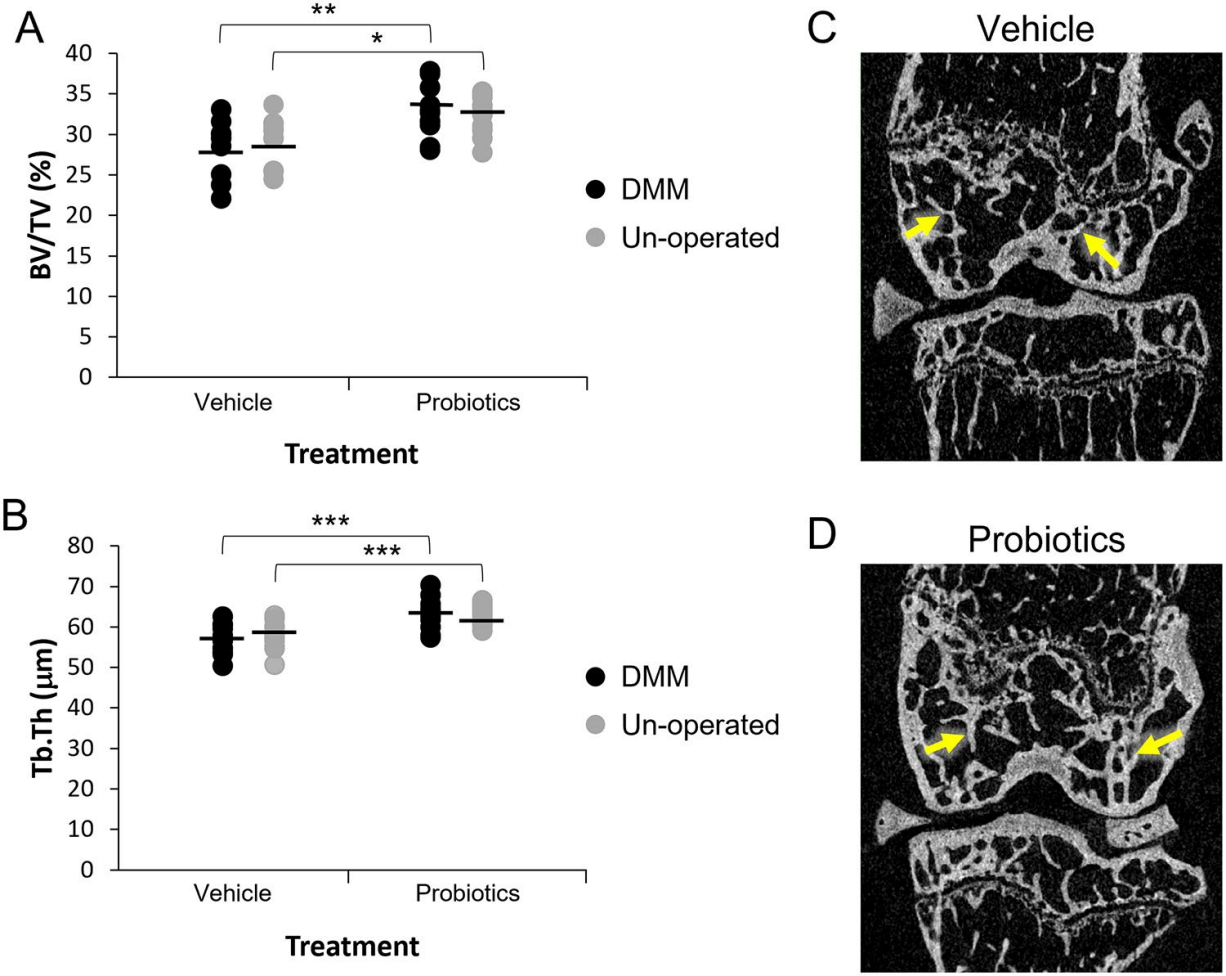
Probiotics increase femoral epiphyseal trabecular bone volume and trabecular thickness. **A** Femoral epiphyseal trabecular bone volume (BV/TV, %) in DMM operated and un-operated knee joints in the two different treatment groups, vehicle (*n* = 10) and probiotics (*n* = 11). **B** Femoral trabecular thickness (Tb.Th, μm) from the same experiment. Each horizontal line in **A** and **B** indicates the mean value for each respective treatment group and the circles represent individual values. *p < 0.05; **p < 0.01; ***p < 0.005, between groups. **C** Representative microCT image from a DMM operated mouse in the vehicle group. **D** representative microCT image from a DMM operated mouse in the probiotics group. The trabecular thickness in subchondral bone is noticeably greater in the probiotics-treated animal versus the vehicle-treated animal (yellow arrows)

The effects of the interventions on femoral subchondral bone are summarized in Table 1 and shown graphically in Fig. 3. In the DMM operated knee, trabecular bone volume (BV/TV) and trabecular thickness (Tb.Th) were both significantly higher in the FMT and probiotics group compared with the control group (Fig. 3A and B). In keeping with this, trabecular pattern factor (Tb.Pf) was significantly lower in the probiotics group compared with the control group (Table 1). The same findings were also observed in the un-operated knee (Fig. 3 and Table 1). The lateral plate thickness of subchondral bone was also higher in the probiotics group compared to the control group in the DMM operated knee but not in the un-operated knee (Table 1).

**Table 1:** MicroCT analysis of femoral subchondral bone from DMM-operated and un-operated knee joints of mice subjected to vehicle (*n* = 10) or probiotics (*n* = 11) treatment

	DMM knee		Un-operated knee	
mCT parameter	Vehicle	Probiotics	Vehicle	Probiotics
BV/TV (%)	28.4 ± 1.1	32.8 ± 1.0*	29.2 ± 1.0	32.4 ± 0.7*
Tb.Th (μm)	57.0 ± 1.2	63.2 ± 1.2***	57.7 ± 1.2	62.6 ± 0.7***
Tb.N (1/mm)	5.0 ± 0.1	5.2 ± 0.1	5.1 ± 0.1	5.2 ± 0.1
Tb.Sp (μm)	183.1 ± 5.3	179.7 ± 3.7	187.0 ± 3.0	180.8 ± 3.1
Tb.Pf (1/mm)	7.2 ± 0.6	4.6 ± 0.6**	6.0 ± 0.7	4.0 ± 0.6*
Medial plate thickness (μm)	95.5 ± 3.4	101.6 ± 2.8	93.8 ± 4.0	95.6 ± 1.7
Lateral plate thickness (μm)	114.4 ± 2.8	125.4 ± 2.4**	118.8 ± 2.5	111.3 ± 4.0

*mCT* microCT, *BV/TV* trabecular bone volume, *Tb.Th* trabecular thickness, *Tb.N* trabecular number, *Tb.Sp* trabecular separation, *Tb.Pf* trabecular pattern factor. Values are mean ± SEM. *p < 0.05; **p < 0.01; ***p < 0.05 *vs.* vehicle

The results of MicroCT analysis of tibial subchondral bone are shown in supplementary Table 1. The findings in tibial subchondral bone were similar to those in femoral subchondral bone with higher values for BV/TV and Tb.Th and lower values for Tb.Pf in the FMT and probiotics group compared with the control group. However, the differences were significant only for the un-operated knee (Supplementary Table 1). Medial plate thickness was significantly higher in the probiotics group compared with the vehicle group in both the DMM operated and non-operated knees, whereas lateral plate thickness was significantly higher only in the un-operated knees.

### Effect of Microbiome Depletion and Probiotics on Joint Inflammation Scores

Inflammation scores were very low in both treatment groups with no significant difference between the groups in the DMM operated knees (Supplementary Fig. 1).

### Effect of Microbiome and Probiotics on Circulating Inflammatory Cytokines

Analysis of serum samples by the MILLIPLEX® MAP Mouse Cytokine/Chemokine Magnetic Bead Panel showed no clear differences across the two intervention groups (Supplementary Table 2).

## Discussion

The gut microbiome consists of 500–1000 different species of micro-organisms which collectively express at least 100-fold more genes than are present the human genome [17]. There is accumulating evidence that disturbances to the gut microbiota contribute to a wide range of inflammatory and metabolic diseases [18]. Over recent years there has been an increasing interest on the role of the microbiome in the pathogenesis of bone and joint disease [19]. For example, a study by Schott et al. showed that obesity-related dysbiosis of the gut microbiome leads to osteoarthritis of obesity, in association with chronic low-grade systemic inflammation [4]. Furthermore, a 16S rRNA gene sequencing profiling study of stool microbiomes by Wang and colleagues, identified seven biomarkers which were associated with an increased risk of OA in overweight individuals [20].

An observational study by Boer et al., looking at the gut microbial composition of 1427 participants, showed that the composition of the gastrointestinal microbiome was associated with knee pain and low-grade inflammation of the knee that was independent of obesity [3]. Similarly, a population-based study by Wei et al. showed that alterations in the composition of the gut microbiome were associated with prevalent symptomatic hand OA [21] and a case–control study by Chen et al. reported that elderly females with OA had significant alterations in the gut microbial composition and function compared to controls [22].

It has previously been reported that administration of *Lactobacillus acidophilus* can reduce the levels of pro-inflammatory cytokines, reduce pain, and improve histopathologic scores in the monoiodoacetate (MIA) experimental model of OA [23], and that *Lacticaseibacillus rhamnosus* ameliorates OA progression by inhibiting joint pain and inflammation [24]. Improvement of OA symptoms have also been reported with probiotics in clinical studies. A randomised double-blind, placebo-controlled clinical trial assessing the effect of *Lacticaseibacillus casei Shirota* (LcS) in patients with knee osteoarthritis reported that knee pain improved and circulating levels of C-reactive protein decreased, leading the authors to conclude that LcS consumption could improve the outcome of knee OA, by reducing inflammatory responses [6].

Here we examined the role of the microbiome in the development of a preclinical model of knee OA [12] and also assessed whether administration of probiotics could influence this process. We chose the destabilisation of medial meniscus (DMM) model of OA because this is thought to reflect the OA that results from knee injury in humans [25]. Our results indicate that the gut microbiome may contribute to the pathogenesis of OA in this model. We found that reconstitution of the microbiome coupled with administration of probiotics protected against cartilage damage due to osteoarthritis at the medial femoral condyle compartment of the joint. These effects were accompanied by changes in subchondral bone, also most marked at the femoral condyle where trabecular bone volume, trabecular thickness and subchondral plate thickness were higher in the probiotics group compared with the control group. Similar changes were observed in tibial compartment, but these were less marked than at the distal femur and were most evident in the un-operated knee. While no significant differences between groups were observed in other joint compartments, this is not unexpected given that OA starts as a focal disease which in this case was closest to the medial site of mechanical destabilisation.

Interestingly, DMM did not cause subchondral bone alterations commonly associated with osteoarthritis, such as thickening of the subchondral bone plate or increased BV/TV in the subchondral region [26]. The reason for this is unclear but could be related to the intestinal dysbiosis cause by the antibiotic treatment which preceded DMM, and which has been reported to cause uncoupling of osteoclastic and osteoblastic activity, leading to reduced trabecular bone volume [27]. It was of interest however, that probiotic treatment increased subchondral trabecular bone volume, trabecular thickness and subchondral plate thickness whether or not DMM had been performed.

Previous studies have suggested that the beneficial effects of probiotics in knee OA might be due to suppression of inflammation [6, 28], but we found no clear evidence to support this. Histological assessment showed that there was very little inflammatory response with average scores for each parameter less than one with no differences between the groups. Consistent with this, no significant difference between groups was observed in circulating concentrations of inflammatory cytokines and chemokines. It is likely that the lack of differences may be due to the fact that OA associated with DMM is mainly due to biomechanical factors as opposed to low grade joint inflammation. In this regard it should be noted that while biomechanical instability can cause OA, it is a multifactorial disease in which other factors are also operative including the effects of genetic predisposition [29] and adipokines released from fat tissue in obese individuals [30].

The positive effects of probiotics on subchondral bone are of interest in the light of previous studies by Li and colleagues who found that, twice-weekly probiotic supplementation of the indigenous microbiota of sex-steroid deficient mice with *Lacticaseibacillus rhamnosus* provided protection against bone loss, whereas supplementation with a non-probiotic strain of *Escherichia coli* did not [31]. The same group of investigators reported that butyrate produced by the intestinal microbiota was required for bone formation induced by parathyroid hormone [32]. Others have implicated IGF1 as another factor produced by the microbiota which may enhance bone formation [33].

The molecular mechanisms by which supplementation with the probiotic strains used in this study protected against the development of cartilage damage and modified subchondral bone are at present unclear and more research will be required to explore the pathways responsible. Despite this caveat, the novel findings presented here indicate that probiotics have the potential to exert a disease modifying effect in OA and indicate the need for further studies in preclinical models and clinically to explore the therapeutic potential of this intervention in a disease that currently has no effective treatment options.

## Supplementary Information

Below is the link to the electronic supplementary material.

Supplementary file1 (PDF 307 KB)

Supplementary file2 (PDF 1225 KB)

Supplementary file3 (PDF 1225 KB)

Supplementary file4 (PDF 1225 KB)

Supplementary file5 (PDF 1225 KB)

Supplementary file6 (PDF 1226 KB)
