# Supplementary material for: Probiotics Inhibit Cartilage Damage and Progression of Osteoarthritis in Mice
Source: Calcif Tissue Int. 2022 Oct 19;112(1):66–73. doi: 10.1007/s00223-022-01030-7 (PMC9813193; doi:10.1007/s00223-022-01030-7)
Supplement: Supplementary file 1 — Supplementary file1 (PDF 307 KB) [file 223_2022_1030_MOESM1_ESM.pdf]

Probiotics inhibit cartilage damage and progression of osteoarthritis in mice

Supplementary Material

Antonia Sophocleous[1,2], Asim Azfer[2], Carmen Huesa[3], Eleni Stylianou[1], Stuart H. Ralston[2]

[1]Department of Life Sciences, School of Sciences, European University of Cyprus, Nicosia, Cyprus
[2]Rheumatology and Bone Diseases Unit, Centre for Genomic and Experimental Medicine, MRC Institute of Genetics and Cancer, Western General Hospital, University of Edinburgh, Edinburgh, UK
[3]Institute of Infection, Immunity & Inflammation, College of Medical, Veterinary and Life Sciences, University of Glasgow, Glasgow, UK

Supplementary Table 1. MicroCT analysis of tibial subchondral bone from DMM-operated and un-operated knee joints of mice subjected to vehicle (n = 10) or probiotics (n = 11) treatment

	DMM knee		Un-operated knee	
**mCT parameter**	**Vehicle**	**Probiotics**	**Vehicle**	**Probiotics**
**BV/TV (%)**	29.4 ± 1.2	32.3 ± 0.8	27.9 ± 1.1	31.6 ± 0.6**
**Tb.Th (μm)**	56.9 ± 1.6	60.5 ± 1.0	53.9 ± 1.2	58.9 ± 0.9***
**Tb.N (1/mm)**	5.2 ± 0.1	5.3 ± 0.1	5.2 ± 0.1	54 ± 0.1
**Tb.Sp (μm)**	171.1 ± 3.9	168.0 ± 2.8	174.3 ± 3.4	169.1 ± 2.2
**Tb.Pf (1/mm)**	10.9 ± 1.0	9.3 ± 0.5	11.4 ± 0.7	9.0 ± 0.5*
**Medial plate thickness (μm)**	93.7 ± 3.9	110.4 ± 3.5**	91.0 ± 3.7	101.9 ± 1.9*
**Lateral plate thickness (μm)**	86.1 ± 2.3	86.8 ± 2.1	85.1 ± 4.3	98.5 ± 2.2*

mCT, microCT; BV/TV, trabecular bone volume; Tb.Th, trabecular thickness; Tb.N, trabecular number; Tb.Sp, trabecular separation; Tb.Pf, trabecular pattern factor. Values are mean ± SEM. *p < 0.05; ** p < 0.01; ***p < 0.005 *vs.* vehicle.

Supplementary Table 2. Cytokine and chemokine concentrations in vehicle- and probiotic-treated groups

Cytokine	Luminex® Region	N	Vehicle	Probiotics
G-CSF	13	7-12	245.5 ± 40.8	323.7 ± 58.6
GM-CSF	15	-	*ND*	*ND*
IFNγ	19	-	*ND*	*ND*
IL-1α	21	8-12	1313.7 ± 377.0	2418.2 ± 590.8
IL-1β	25	-	*ND*	*ND*
IL-2	26	-	*ND*	*ND*
IL-4	28	-	*ND*	*ND*
IL-5	30	6-12	11.2 ± 1.4	13.4 ± 1.7
IL-6	34	4-8	4.4 ± 0.9	6.4 ± 1.4
IL-7	36	-	*ND*	*ND*
IL-9	38	7-11	102.4 ± 15.3	102.3 ± 11.4
IL-10	43	8-11	17.4 ± 2.5	10.5 ± 2.2
IL-12 (p40)	45	5-9	9.1 ± 2.0	11.4 ± 2.3
IL12 (p70)	47	-	*ND*	*ND*
IL-13	52	7-11	40.5 ± 2.2	42.4 ± 2.2
IL-15	54	6-10	112.2 ± 17.9	96.2 ± 20.7
IL-17	56	7-12	16.4 ± 2.6	13.6 ± 1.4
IP-10	57	7-10	208.2 ± 26.9	211.9 ± 14.3
KC	61	7-11	123.9 ± 12.5	104.4 ± 12.9
MCP-1	62	7-12	38.5 ± 5.4	27.4 ± 4.2
MIP-1α	64	7-11	82.1 ± 10.1	71.7 ± 6.5
MIP-1β	66	8-11	67.6 ± 5.6	57.3 ± 5.3
MIP-2	73	8-11	257.1 ± 25.2	296.3 ± 33.3
RANTES	75	8-12	55.6 ± 5.0	45.1 ± 6.5
TNFα	77	-	*ND*	*ND*

G-CSF = Granulocyte colony stimulating factor; GM-CSF = granulocyte-macrophage colony stimulating factor; IL = Interleukin; KC = keratinocyte chemoattractant chemokine; MCP1 = monocyte chemoattractant protein 1; MIP = Macrophage inflammatory protein; RANTES = Regulated on activation normal T cell expressed and secreted; TNF = Tumour necrosis factor. Values are mean ± SEM from 4-12 mice per group and the cytokine concentrations are in pg/ml. N = number of samples assayed; ND = not detectable. There was no significant difference between the groups considering multiple testing.

Supplementary Figure 1. Joint inflammation scores in the different treatment groups

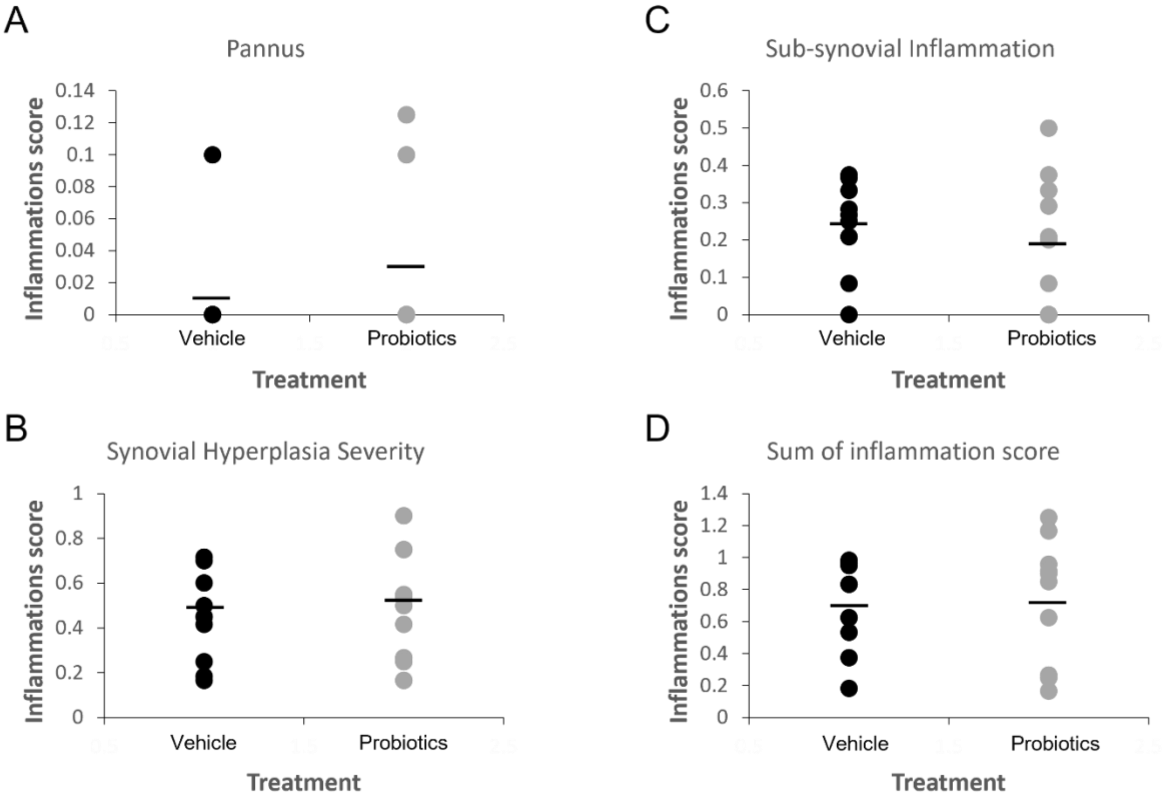

Local inflammation in DMM-operated knee joints of mice subjected to the two different treatment groups, vehicle (n = 10) and probiotics (n = 11). **Panel A.** Pannus formation, defined as fibrous tissue/synovium/ inflammatory cell outgrowth spreading over the surface of the bone and/or cartilage at the joint margins; **Panel B.** Synovial hyperplasia, which includes the score for the maximum hyperplasia seen anywhere along the area assessed; **Panel C.** Sub-synovial inflammation, where the infiltration of inflammatory cells (neutrophils, macrophages and/or lymphocytes) is evaluated; **Panel D.** Sum inflammation score of A, B and C. The horizontal line indicates the mean value for each treatment group.
